# Net protein and metabolizable protein requirements for maintenance and growth of early-weaned Dorper crossbred male lambs

**DOI:** 10.1186/s40104-017-0172-6

**Published:** 2017-05-01

**Authors:** Tao Ma, Kaidong Deng, Yan Tu, Naifeng Zhang, Bingwen Si, Guishan Xu, Qiyu Diao

**Affiliations:** 1 Feed Research Institute/Key laboratory of Feed Biotechnology of the Ministry of Agriculture, Chinese Academy of Agricultural Sciences, Beijing, China; 2 College of Animal Science, Jinling Institute of Technology, Nanjing, Jiangsu China; 3 College of Animal Science, Tarim University, Alar, Xinjiang China

**Keywords:** Growth, Lamb, Maintenance, Metabolizable protein, Net protein

## Abstract

**Background:**

Dorper is an important breed for meat purpose and widely used in the livestock industry of the world. However, the protein requirement of Dorper crossbred has not been investigated. The current paper reports the net protein (NP) and metabolizable protein (MP) requirements of Dorper crossbred ram lambs from 20 to 35 kg BW.

**Methods:**

Thirty-five Dorper × thin-tailed Han crossbred lambs weaned at approximately 50 d of age (20.3 ± 2.15 kg of BW) were used. Seven lambs of 25 kg BW were slaughtered as the baseline animals at the start of the trial. An intermediate group of seven randomly selected lambs fed ad libitum was slaughtered at 28.6 kg BW. The remaining 21 lambs were randomly divided into three levels of dry matter intake: ad libitum or 70% or 40% of ad libitum intake. Those lambs were slaughtered when the lambs fed ad libitum reached 35 kg BW. Total body N and N retention were measured.

**Results:**

The daily NP and MP requirements for maintenance were 1.89 and 4.52 g/kg metabolic shrunk BW (SBW^0.75^). The partial efficiency of MP utilization for maintenance was 0.42. The NP requirement for growth ranged from 12.1 to 43.5 g/d, for the lambs gaining 100 to 350 g/d, and the partial efficiency of MP utilization for growth was 0.86.

**Conclusions:**

The NP and MP requirements for the maintenance and growth of Dorper crossbred male lambs were lower than the recommendations of American and British nutritional systems.

## Background

In the intensive livestock industry, protein is commonly the most expensive feed component and therefore, it is necessary to have a precise understanding of protein requirement of livestock not only to ensure farm profitability, but also to help reduce nitrogen (N) emission to the environments [1]. Modern feeding systems, such as Agricultural and Food Research Council (AFRC) [2], Commonwealth Scientific and Industrial Research Organisation (CSIRO) [3], and National Research Council (NRC) [4], have reported protein and other nutrient requirements of sheep, which are widely adopted for diet formulation in the world.

The Dorper is a popular sire breed for meat production in both South Africa and United States [5]. In recent years, Dorper sheep have been imported to China to improve the growth performance and carcass traits of indigenous breed, among which the thin-tailed Han sheep is one of the most famous native breeds with high prolificacy, as it carries mutations in both the *BMPR-1B* and *BMP15* genes [6]. Thus, the Dorper × thin-tailed Han crossbreed has become one of the dominant breeds for lamb meat production.

Our research team conducted a series of studies on the nutrient requirements (energy, protein, and minerals) of Dorper × thin-tailed Han crossbred sheep [7–9]. In this paper, we reported the protein requirements of male lambs after weaning ranging from 20 to 35 kg BW by the comparative slaughter technique.

## Methods

The current research was conducted from June to September 2011 at the Experimental Station of the Chinese Academy of Agricultural Sciences (CAAS), Nankou (40°22′N, 116°1′E), Beijing, China. The animals were kept in an enclosed animal house and the mean minimum and maximum room temperatures observed during the experimental period were 15.5 and 26.5 °C (average 21.0 °C), respectively.

### Animals, treatments, and experimental procedure

Thirty-five Dorper × thin-tailed Han crossbred male lambs weaned at approximately 50 d of age with 20.4 ± 2.15 kg BW were used in a completely randomized design to measure protein requirements for maintenance and growth. The experimental diet was formulated according to NRC [4] with a concentrate-to-forage ratio of 44:56 on a dry matter (DM) basis (Table 1). The lambs with ad libitum intake were fed once daily at 0800 h and allowed 10% of refusals. The amount of feed provided to the restricted feed intake groups was adjusted daily based on the average DM intake (DMI) of the ad libitum group from the previous day. Feed and refusals were sampled daily and frozen at −20 °C until analysis.

**Table 1:** Ingredient and chemical compositions of the pelleted mixture diet

Items	Value, %DM
Ingredients, DM basis	
Milled Chinese wildrye hay	55.0
Cracked corn grain	29.4
Soybean meal	14.0
Dicalcium phosphate	0.86
Salt	0.50
Mineral/vitamin premix^a^	0.24
Chemical composition	
ME, MJ/kg DM	8.89
DM, % as fed	95.5
CP, % of DM	11.9
EE, % of DM	2.71
Ash, % of DM	6.32
NDF, % of DM	40.9
ADF, % of DM	15.2
Calcium, % of DM	0.68
Phosphorus, % of DM	0.33

^a^Manufactured by Precision Animal Nutrition Research Centre, Beijing, China. The premix contained (per kg): 22.1 g Fe, 13.0 g Cu, 30.2 g Mn, 77.2 g Zn, 19.2 g Se, 53.5 g I, 9.10 g Co, 56.0 g vitamin A, 18.0 g vitamin D_3_, and 170 g vitamin E

A comparative slaughter trial was conducted as described previously [9]. Briefly, the initial body composition was measured on seven lambs slaughtered at 20 kg BW (baseline group). An intermediate slaughter group with seven randomly selected lambs fed ad libitum were slaughtered at 28.6 kg BW. The remaining 21 lambs were randomly assigned to three levels of DMI: ad libitum or 70% or 40% of ad libitum intake. Thus, the lambs were pair-fed in seven slaughter groups, with each group consisting of one lamb from each level of intake. When the lambs fed ad libitum of each slaughter group reached 35 kg BW, all lambs within a slaughter group were fasted and slaughtered. Seven lambs in the baseline and intermediate group were slaughtered in one day, respectively, while the remaining 21 lambs were slaughtered in three consecutive days. All lambs were slaughtered by exsanguination after stunning by CO_2_ inhalation. Blood, carcass, head, feet, hide, wool, viscera, and adipose tissue removed from the internal organs were weighed. The empty body weight (EBW) was calculated by subtracting the weight of the digestive tract contents from the shrunk BW (SBW), which was measured as BW after a 16-h fast of feed and water. Carcasses and heads were split longitudinally into two identical halves and the muscle, bone, and fat were dissected from the right-half carcass, head, and feet, while the whole hide and whole viscera were ground and homogenized separately and frozen at −20 °C until analysis. Wool was clipped with electrical clippers after slaughter, and subsamples were collected and stored at 4 °C.

### Chemical analyses

Feed and orts were oven-dried at 55 °C for 72 h, ground to pass through a 1-mm screen for analyzing DM (method 930.15) [10], ash (method 924.05) [10], ether extract (EE) (method 920.85) [10], and nitrogen (N) [11]. The gross energy (GE) of feed was measured with a bomb calorimeter (C200, IKA Works Inc., Staufen, Germany). The neutral detergent fiber (NDF) and acid detergent fiber (ADF) of feed were measured according to Van Soest et al. [12] and Goering and Van Soest [13], respectively. Calcium of feed was analyzed using an atomic absorption spectrophotometer (M9W-700, Perkin-Elmer Corp., Norwalk, CT, USA) (method 968.08) [10]. The total P of feed was analyzed by the molybdovanadate colorimetric method (method 965.17) [10] using a spectrophotometer (UV-6100, Mapada Instruments Co., Ltd., Shanghai, China).

The DM of all body components except wool was determined following lyophilization for 72 h to constant weight. The samples were then analyzed for N as described above. Wool samples were cut into 2-mm pieces with scissors and analyzed for N as described above.

### Data calculations

Metabolizable protein supply: The ratio of metabolizable protein (MP) to OM intake reported in a previous in vivo study (i.e., 69.4, 88.9, and 102.4 g MP/kg OM intake for ad libitum, 70% and 50% of the ad libitum intake, respectively) [14] with Dorper × thin-tailed Han crossbred sheep subjected to the same feeding regime as the present study was used to calculate the individual MP intake (MPI) by the ram lambs.

Prediction of the initial body N content and N retention (NR): The NR in the body of the lambs in the comparative slaughter trial was calculated as the difference between the final and initial body N content. The initial body N content of each animal was calculated from its initial EBW using a regression equation developed from the relationship between the body N content and EBW of the baseline animals (*R* ^2^ = 0.92, root mean square error [RMSE] = 0.011, n = 7, *P* < 0.001): log_10_ empty body N, kg = 1.536 (±0.174) + [0.957 (±0.144) × log_10_ EBW, kg]. The initial SBW of each animal was computed from its initial BW (*R* ^2^ = 0.97, RMSE = 0.280, n = 7): SBW, kg = 0.571 (±1.457) + [0.915 (±0.069) × BW, kg], and the initial EBW of each animal was computed from its initial BW (*R* ^2^ = 0.86, RMSE = 0.429, n = 7): EBW, kg = 2.336 (±2.227) + [0.695 (±0.105) × BW, kg].

Protein requirements for maintenance: A linear regression of daily NR on daily N intake (NI) was used to calculate the net protein (NP) requirement for maintenance. The intercept of the regression was assumed the endogenous and metabolic losses of N multiplied by the factor 6.25, which is assumed the maintenance requirement for NP (NP_m_, g/kg^0.75^ SBW). The MP required for maintenance (MP_m_, g/kg^0.75^ SBW) was then estimated by regressing the NR on MPI and extrapolating the linear regression to zero NR. The efficiency of MP use for maintenance (k_pm_) was computed as NP_m_/MP_m_.

Protein requirements for growth: The NP requirements for body weight gain (NP_g_) were calculated as the difference between body protein content at different intervals. For example, the NP_g_ of a lamb with 20 kg SBW and 250 g of average daily gain (ADG) was computed as the difference between body protein contents at 20.25 and 20 kg SBW. Body protein contents were predicted from EBW using an allometric equation according to ARC (1980): log_10_ protein, kg = a + [b × log_10_ EBW, kg]. To estimate the partial efficiencies of MP use for body weight gain (k_pg_), a regression model through the origin was used to partition the utilization of MPI above maintenance for body protein retention as follows: MPI_g_ = b × RP, where MPI_g_ (g/kg^0.75^ of SBW) is the MPI above maintenance calculated as the difference between the total MPI and MP_m_, RP (g/kg^0.75^ of SBW) is the daily retention of body protein, and the estimated parameter b is the amount of MP (g) required to retain 1 g of protein, and its inverse was assumed to be k_pg_.

### Statistical analyses

The data were analyzed as a completely randomized design using the SAS statistical software package (version 9.1; SAS Institute, Inc., Cary, NC). Intake, body composition, and growth rate were analyzed using a one-way ANOVA. Pairwise comparisons of means were performed by Tukey’s multiple range tests once the significance of the treatment effect was declared at *P* < 0.05. The statistical model is: *y*_*ij*_ *= μ + α*_*i*_ *+ ε*_*ij*_, where *y*_*ij*_ = dependent variable, *μ* = overall mean of *Yij*, *α*_*i*_ = effect of the diet (i = 1 to 3), and *ε*_*ij*_ = error contribution. Linear regressions were conducted with a GLM, and observations with a studentized residual > 2.5 or < −2.5 were considered outliers. The assumptions of the models, in terms of homoscedasticity, independency, and normality of errors, were examined by plotting residuals against the predicted values.

## Results

The intake of OM and N increased with feeding level (*P* < 0.001; Table 2). The lambs fed with ad libitum intake retained greater N than those with either level of restricted feed intake (*P* < 0.001). The lambs fed at 40% of the ad libitum intake had a lower MPI than those in the other two groups (*P* < 0.001), and the MPI did not differ between lambs fed at ad libitum and 70% of the ad libitum intake (*P* = 0.286).

**Table 2:** Daily protein intake of Dorper × thin-tailed Han crossbred ram lambs at ad libitum (AL) or restricted to 70% or 40% of AL intake^1^

	Level of feed intake^2^			SEM	*P* ^4^
Items	AL	70%	40%		
ADG, g	324.2^a^	189.1^b^	37.6^c^	19.3	<0.001
Initial SBW, kg	19.3	19.5	18.5	0.22	0.271
Final SBW, kg	29.9^a^	27.8^a^	20.4^b^	0.91	<0.001
OM intake, g/d	107.6^a^	84.2^b^	53.3^c^	4.37	<0.001
N intake, g/(kg SBW^0.75^ · d)	1.83^a^	1.43^b^	1.01^c^	0.08	<0.001
N retention, g/(kg SBW^0.75^ · d)	0.47^a^	0.29^b^	0.11^c^	0.03	<0.001
MP intake, g/(kg SBW^0.75^ · d)^3^	7.47^a^	7.49^a^	5.46^c^	0.18	<0.001

^1^ADG: average daily gain; SBW: shrunk body weight; MP: metabolizable protein

^2^Ad libitum or restricted to 70% or 40% of ad libitum of an identical diet

^3^Calculated from the ratio of MP supply to OM intake reported by Ma et al. [14]

^4^ ^a,b,c^Means bearing different superscripts differ (*P* < 0.05)

**Fig. 1:**
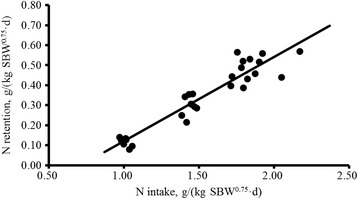
Relationship between N retention (NR) and N intake (NI) of Dorper × thin-tailed Han crossbred male lambs from 20 to 35 kg of BW. NR, g/kg SBW^0.75^ = −0.303 (±0.046) + [0.422 (±0.040) × NI, g/kg of SBW^0.75^], *R* ^2^ = 0.89, RMSE = 0.054, n = 28

Figure 1 shows the linear relationship between NR and NI: NR, g/(kg SBW^0.75^ · d) = −0.303 (±0.046) + [0.422 (±0.040) × N intake, g/(kg SBW^0.75^ · d)] (*R* ^2^ = 0.89, RMSE = 0.054, n = 28, *P* < 0.001). The endogenous and metabolic loss of N, estimated as the intercept of the linear regression, was 303 ± 46 mg/kg^0.75^ SBW, which corresponds to an NP_m_ of 1.89 ± 0.29 g/kg^0.75^ SBW.

**Fig. 2:**
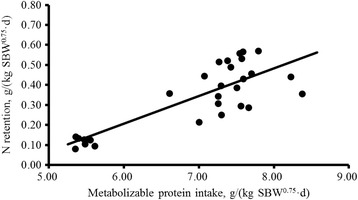
Relationship between N retention (NR) and metabolizable protein intake (MPI) of Dorper × thin-tailed Han crossbred male lambs from 20 to 35 kg of BW. NR, g/kg SBW^0.75^ = − 0.628 (±0.138) + [0.139 (±0.020) × MPI, g/kg SBW^0.75^], *R* ^2^ = 0.70, RMSE = 0.096, n = 28

Figure 2 shows the linear relationship between NR and MPI: NR, g/(kg SBW^0.75^ · d) = − 0.628 (±0.138) + [0.139 (±0.020) × MPI, g/(kg SBW^0.75^ · d)], *R* ^2^ = 0.70, RMSE = 0.096, n = 28, *P* < 0.001). The MP required for maintenance by extrapolating the linear regression to zero N retention was 4.52 g/kg^0.75^ SBW. Consequently, the k_pm_ was 0.42 for Dorper × thin-tailed Han crossbred male lambs from 20 to 35 kg of BW.

The partial efficiencies of MP use for body weight gain were estimated using a regression model and assuming that the MPI above maintenance (MPI_g_) is partially recovered as body protein for growth (PR_g_, g/kg^0.75^ of SBW). The regression equation was: PR_g_, g/(kg^0.75^ SBW · d) = 0.002(±0.052) + [0.864±0.123) × MPI_g_, g/(kg^0.75^ of SBW · d)] (*R* ^2^ = 0.70, n = 28, RMSE = 0.081). The slope of the regression equation (0.86) represents k_pg_.

The allometric equation between body protein and EBW (*R* ^2^ = 0.96; RMSE = 0.012; n = 20) of the ram lambs with free consumption of feed was proposed as log_10_ protein, g = 2.385 (±0.072) + [0.922 (±0.054) × log_10_ EBW, kg]. The NP_g_ and MP for growth (MP_g_) were therefore calculated accordingly (Tables 3 and 4).

**Table 3:** Requirements of net protein (NP) for growth (g/d) of Dorper × thin-tailed Han crossbred male lambs from 20 to 35 kg of body weight (BW)

ADG, g/d	BW, kg			
	20	25	30	35
100	12.4	12.3	12.2	12.1
200	24.9	24.6	24.4	24.2
300	37.3	36.9	36.6	36.3
350	43.5	43.0	42.7	42.3

ADG: average daily gain

**Table 4:** Requirements of metabolizable protein (MP) for growth (g/d) of Dorper × thin-tailed Han crossbred male lambs from 20 to 35 kg of body weight (BW)

ADG, g/d	BW, kg			
	20	25	30	35
100	14.4	14.3	14.2	14.1
200	29.0	28.6	28.4	28.1
300	43.4	42.9	42.6	42.2
350	50.6	50.0	49.7	49.2

ADG: average daily gain

## Discussion

The NP_m_ is the quantity of protein to sustain tissue proteins by counterbalancing the inevitable losses of urinary, fecal, and dermal N [3]. AFRC [15] suggested daily endogenous and metabolic N losses of 350 mg/kg BW^0.75^ for lambs nourished by intra-gastric infusions. However, this method might overestimate the endogenous N requirement due to the lack of conservation of protein by the microbial capture of N. CSIRO [3] suggested the NP_m_ of the sheep is the sum of the endogenous urinary loss (0.147 × BW + 3.375) and fecal loss (15.2 g/kg DM intake). Thus, the NP_m_ for a lamb of 28 kg consuming 1.02 kg of DM daily (this is the average DM intake of the 28 lambs used in the current study) is about 23.0 g, which is 10% higher than the current value (1.72 g/kg BW^0.75^). In the current study, the NP_m_ calculated by partial regression NR on NI (1.72 g/kg BW^0.75^ or 1.89 g/kg SBW^0.75^) is slightly higher than that of the Ile de France (1.56 g/kg SBW^0.75^) [16] and Texel crossbreeds (1.52 g/kg SBW^0.75^) [17], but lower than that of Morada Nova (1.83 g/kg BW^0.75^) [18] male lambs measured using the same methods. The lambs fed at the maintenance level had similar NR (around 0.1 to 0.2 g/kg of SBW^0.75^) among all these studies. However, when fed higher than the maintenance level, Dorper crossbred had a relatively higher NR (0.25 to 0.65 g/kg of SBW^0.75^) than the Ile de France [16] and Texel crossbreeds [17], but a lower NR than Morada Nova lambs (0.25 to 1.0 g/kg of BW^0.75^) [18]. Therefore, the variations in NP_m_ can be explained by the differential utilization efficiency of protein or amino acids by the tissues during the growth of lambs. On the other hand, the above studies were all conducted in a tropical or sub-tropical zone with high humidity, while the current study was conducted in a warm temperate zone where the weather is always dry during summer. As reviewed by Marai et al. [19], both temperature and humidity could have influence on nutrient digestibility and degradation in the rumen; thus, the experimental condition could be another important factor that contributes to the differences in NP_m_ apart from methods or animal breeds. We concluded that 1.69 g/kg BW^0.75^ or 1.81 g/kg SBW^0.75^ is suitable for the NP_m_ of Dorper crossbred male lambs from 20 to 35 kg of BW.

The MP_m_ obtained in the current study was 4.52 g/kg SBW^0.75^. When scaled to BW, our value (4.37 g/kg BW^0.75^) was close to Liu et al. [20], who found an MP_m_ of 4.41 g/kg BW^0.75^ from a linear multiple regression of MP requirements against the live weight, live weight gain, and wool growth of sheep (n = 213) in a feeding study. Nevertheless, our result is greater than that suggested by AFRC (2.19 g/kg BW^0.75^) [2], INRA (2.50 g/kg BW^0.75^) [21], or NRC (3.72 g/kg BW^0.75^) [22]. In a more recent study, where a comparative slaughter trial was also used, a MP_m_ of 2.31 g/kg SBW^0.75^ was observed in Texel crossbred lambs [17]. The methods adopted by UK [2], Australia [3], USA [4], and France [21] were all based on a common overall model, although requirements were expressed in different terms. In the current study, MP was calculated based on the method reported by Ma et al. [14], who conducted an in vivo study and measured MP using sheep with ruminal and duodenal cannula. Therefore, the discrepancy in the calculation of MP is inevitably associated with the methodologies adopted. As there is still a lack of simple and robust methods for calculating MP, this area requires further investigation and examination.

The NP_g_ values (12.4, 24.9, and 37.3 g/d) determined in the current study are extensively lower than those of early maturing growing lambs (23.5, 30.5, and 50.0 g/d) of 20 kg BW gaining 100, 200, and 300 g/d recommended by NRC [4], assuming a k_pg_ of 0.50. AFRC [2] used two equations proposed by ARC [23], NP_f_ (g/d) = ADG × (160.4 – 1.22 × BW + 0.0105 × BW^2^) and NP_w_ (g/d) = 3 + 0.1 × NP_f_, where NP_f_ is the NP requirement for fleece-free body growth and NP_w_ is the NP requirement for wool growth, to predict the protein requirement for the growth of body and fleece in lambs, respectively. Using those equations, the NP_g_ (NP_f_ + NP_w_) was approximately 26% to 49% higher (range from 17.4 to 57.0 g/d) than values determined in the current study. Therefore, caution should be taken before applying certain evaluation systems to avoid the overestimation of NP_g_ of Dorper crossbred lambs. By using the same method, our NP_g_ results were 26% higher than those of the Texel crossbreed [17] growing from 20 to 35 kg of SBW gaining 100 and 200 g/d, but 20% lower than that of Morada Nova lambs [18] growing from 20 to 30 kg of BW gaining 100, 200, and 300 g/d, respectively. Although the ME (0.76 vs. 0.73 MJ/kg SBW^0.75^), NI (2.12 vs. 2.20 g/kg SBW^0.75^), and N retention/N intake (26.1% vs. 25.9%) were close, the ADG of ad libitum groups were much higher for the Dorper crossbred (324 g) than Texel crossbred (245 g) lambs reported by Galvani et al. [17]. Many factors could be associated with such discrepancies, including breed, physiological stage, and experimental conditions.

The partial efficiency of use of MP_m_ for NP_m_ (k_pm_) was calculated to be 0.42 in the present study. This value is lower than previously adopted 1.0 by AFRC [15] or 0.67 by CSIRO [3], as well as lower than that of Texel crossbred lambs (0.66) [17]. The partial efficiency of use of MP_g_ for NP_g_ (k_pg_) obtained in the current study (0.86) was greater than that adopted by AFRC (0.59) [2], CSIRO (0.70) [3] and by Galvani et al. [17] in Texel crossbred lambs (0.71). Those discrepancies could be attributed to animal factors, including breed, maturity, and physiological status. The method for calculating or determining MP may be another factor contributing to the variability of the efficiency of MP use. NRC [4] suggested a simple equation, MP = CP × (0.64 + 0.16 × % UDP)/100, where UDP is undegraded dietary protein, to convert CP to MP. By using this equation, MP/CP was 0.72 in all treatments, and k_pm_ and k_pg_ were 0.59 (1.89/3.22) and 0.58, respectively. Our previous study showed that a decreased feed intake could increase total-tract N digestibility without affecting ruminal N degradability [24], and the increased duodenal N digestibility could be due to the prolonged gastric empty time. Thus, it could not be expected that MP/CP were identical under a different feeding level. In the current study, MP was calculated from OM intake based on the results of our previous study using 6-month-old Dorper × thin-tailed Han male lambs (41.3 ± 2.8 kg BW) with both ruminal and duodenal cannula fed at three different levels (ad libitum, 70%, and 50% of ad libitum) in which MP supply/OM intake (g/kg) was 69.4, 88.9, and 102.4, respectively. As there is still lack of a simple method for the calculation of MP, this could be a reasonable way to calculate MP in the current study. Nevertheless, considering the difference in animal physiology status (BW, age, and cannulation) between our previous and current studies, further study is still needed to examine the utilization efficiency of MP for both the maintenance and growth for early-weaning lambs.

## Conclusions

In conclusion, the current study suggested that the protein requirements for the maintenance and growth of Dorper × thin-tailed Han early-weaned crossbred male lambs were lower than the recommendations of AFRC (1993) and NRC (2007).

## Abbreviations

ADF: Acid detergent fiber
ADG: Average daily gain
CP: Crude protein
DM: Dry matter
EBW: Empty body weight
EE: Ether extract
GE: Gross energy
ME: Metabolizable energy
MNI_g_: Metabolizable N intake above maintenance
MP: Metabolizable protein
MP_g_: MP requirements for body weight gain
MPI _g_: MP intake above maintenance
MP_m_: Metabolizable protein for maintenance
N: Nitrogen
NDF: Neutral detergent fiber
NP: Net protein
NP_f_: Net protein requirements for fleece-free body growth
NP_g_: Net protein requirements for body weight gain
NP_m_: Net protein for maintenance
NP_w_: Net protein requirements for wool growth
NR: Nitrogen retention
OM: Organic matter
RMSE: Root mean square error
RN_g_: Body N for growth
SBW: Shrunk body weight
UDP: Undegraded dietary protein

## References

[1] Ma T, Xu GS, Deng KD, Ji SK, Tu Y, Zhang NF (2016). Energy requirements of early-weaned Dorper cross-bred female lambs. J Anim Physiol An N.

[2] AFRC (1993). Energy and protein requirements of ruminants. An advisory manual prepared by the Agricultural and Food Research Council Technical Committee on Responses to Nutrients.

[3] CSIRO (2007). Nutrient requirements of domesticated ruminants.

[4] NRC (2007). Nutrient Requirements of Small Ruminants: Sheep, Goats, Cervids, and New World Camelids.

[5] Snowder GD, Duckett SK (2003). Evaluation of the South African Dorper as a terminal sire breed for growth, carcass, and palatability characteristics. J Anim Sci.

[6] Chu MX, Liu ZH, Jiao CL, He YQ, Fang L, Ye SC (2007). Mutations in *BMPR-IB* and *BMP-15* genes are associated with litter size in Small Tailed Han sheep (*Ovis aries*). J Anim Sci.

[7] Deng KD, Diao QY, Jiang CG, Tu Y, Zhang NF, Liu J (2012). Energy requirements for maintenance and growth of Dorper crossbred ram lambs. Livest Sci.

[8] Deng KD, Jiang CG, Tu Y, Zhang NF, Liu J, Ma T (2014). Energy requirements of Dorper crossbred ewe lambs. J Anim Sci.

[9] Xu GS, Ma T, Ji SK, Deng KD, Tu Y, Jiang CG (2015). Energy requirements for maintenance and growth of early-weane d Dorper crossbred male lambs. Livest Sci.

[10] AOAC (1990). Official methods of analysis.

[11] Marshall CM, Walker AF (1978). Comparison of a short method for Kjeldahl digestion using a trace of selenium as catalyst, with other methods. J Sci Food Agri.

[12] Van Soest PJ, Robertson JB, Lewis BA (1991). Methods for dietary fiber, neutral detergent fiber and non-starch polysaccharides in relation to animal nutrition. J Dairy Sci.

[13] Goering HG, Van Soest JP. Forage fiber analysis. Agricultural Handbook, vol. 379. Washington: UPSDA; 1970.

[14] Ma T, Deng KD, Tu Y, Zhang NF, Jiang CG, Liu J (2015). Effect of feed intake on metabolizable protein supply in Dorper × thin-tailed Han crossbred lambs. Small Rumin Res.

[15] AFRC (1992). Technical committee on responses to nutrients, Report 9. Nutritive Requirements of Ruminant Animals: Protein. Nutrition Abstracts and Reviews. Series B.

[16] Silva AMA, Silva Sobrinho AG, Trindade IACM, Resende KT, Bakke OA (2003). Net requirements of protein and energy for maintenance of wool and hair lambs in a tropical region. Small Rumin Res.

[17] Galvani DB, Pires CC, Kozloski GV, Sanchez LMB (2009). Protein requirements of Texel crossbred lambs. Small Rumin Res.

[18] Costa MRGF, Pereira ES, Silva AMA, Paulino PVR, Mizubuti IY, Pimentel PG (2013). Body composition and net energy and protein requirements of Morada Nova lambs. Small Rumin Res.

[19] Marai IFM, El-Darawany AA, Fadiel A, Abdel-Hafez MAM (2007). Physiological traits as affected by heat stress in sheep-a review. Small Rumin Res.

[20] Liu SM, Smith TL, Karlsson LJE, Palmer DG, Besier RB (2005). The costs for protein and energy requirements by nematode infection and resistance in Merino sheep. Livest Prod Sci.

[21] INRA (1989). Ruminant nutrition: Recommended allowances and feed tables.

[22] NRC (1987). Predicting feed intake of food-producing animals.

[23] ARC (1980). The Nutrient Requirements of Ruminant Livestock.

[24] Ma T, Deng KD, Jiang CG, Tu Y, Zhang NF, Liu J (2013). The relationship between microbial N synthesis and urinary excretion of purine derivatives in Dorper × thin-tailed Han crossbred sheep. Small Rumin Res.

